# In Vitro Propagation of an Endangered *Helianthus verticillatus* by Axillary Bud Proliferation

**DOI:** 10.3390/plants9060712

**Published:** 2020-06-03

**Authors:** Marzena Nowakowska, Žaklina Pavlović, Marcin Nowicki, Sarah L. Boggess, Robert N. Trigiano

**Affiliations:** 1 Department of Entomology and Plant Pathology, Institute of Agriculture, University of Tennessee, Knoxville, TN 37996, USA; zpavlovi@vols.utk.edu (Ž.P.); mnowicki@utk.edu (M.N.); sbogges1@vols.utk.edu (S.L.B.); rtrigian@utk.edu (R.N.T.); 2 Department of Genetics, Breeding, and Biotechnology of Vegetable Crops, Research Institute of Horticulture, 96-100 Skierniewice, Poland

**Keywords:** Asteraceae, clonal fidelity, cytokinin, nodal explants, micropropagation, whorled sunflower

## Abstract

*Helianthus verticillatus* (Asteraceae), whorled sunflower, is a perennial species restricted to a few locations in the Southeastern United States. Habitat loss has caused *H. verticillatus* to become rare, and since 2014, it has been federally listed as an endangered species. As a part of the recovery plan for the restoration and protection of *H. verticillatus*, an efficient micropropagation protocol based on axillary shoot proliferation was developed. Various concentrations of 6-benzylaminopurine (BAP; 0 to 4.44 µM) were examined for their morphogenetic potential in the regeneration of six genotypes of *H. verticillatus* from the nodal explants derived from greenhouse-grown plants. Both the BAP concentration and genotype had significant effects on the regeneration capacity of *H. verticillatus*. Although the induced buds were observed on ½-strength Murashige and Skoog medium without plant growth regulators, a higher rate of induction and bud development were achieved on media with either 0.88 or 2.22 µM BAP, regardless of the genotype. Successful rooting of the induced shoots was achieved within four weeks after the transfer from the induction medium to the fresh ½-strength MS medium, but the rooting efficiency was dependent on the plant’s genetic background. Regenerated plantlets, with well-developed shoots and roots, were acclimatized successfully to greenhouse conditions with a 97% survival rate. Simple sequence repeats (SSRs) markers were employed to assess the genetic uniformity of the micropropagated plants of *H. verticillatus*. No extraneous bands were detected between regenerants and their respective donor plants, confirming the genetic fidelity and stability of regenerated plants. To our knowledge, the protocol developed in this study is the first such report for this endangered species.

## 1. Introduction

*Helianthus verticillatus* Small, whorled sunflower, is a perennial, self-incompatible species belonging to the Asteraceae [1]. In the natural setting, it propagates clonally by the production of slender rhizomes, as well as by sexual reproduction by seeds if compatible genotypes are present. *Helianthus verticillatus* is an extremely rare species, and found in the following 5 locations: Madison and McNary County, Tennessee, Cherokee Co., Alabama, Floud Co., Georgia, and a new recently discovered location in Mississippi Co., Arkansas [2,3]. Due to loss and degradation of the habitat, *H. verticillatus* was recently listed as a federally endangered species by the U.S. Fish and Wildlife Service [4].

*Helianthus verticillatus* owes its name to a unique verticillate leaf arrangement in whorls of three or four leaves [1]. Vigorous growth (up to four meters tall) and inflorescences with multiple showy, yellow rays of flowers render it a potential ornamental plant. The attractive flowers are a popular native food source for many insect pollinators [5]. Furthermore, the seeds have a high linoleic acid concentration and low saturated fatty acid profile (a reduction of 30% compared with commercial sunflower oil), and thus *H. verticillatus* is one of the possible sources for those traits for the improvement of cultivated sunflower [6,7].

*Helianthus verticillatus* is classified as an endangered species [4], therefore there is a need to develop a strategy to protect and conserve its biodiversity. As a part of this strategy, clonal multiplication methods that employ vegetative propagation via stem cuttings and micropropagation techniques may play a key role in contributing to the conservation of endangered and rare species. Unlike conventional asexual propagation methods, in vitro techniques offer highly effective tools for the rapid multiplication of pathogen-free plantlets in a relatively short time and space, and have the advantage of starting from a minimal amount of plant material with low impact on wild populations [8]. For those reasons, reliable in vitro protocols are well-suited for conservation ex situ of rare and endangered species, and offer a possibility of re-introduction to natural habitats [9,10,11,12,13,14,15,16,17,18,19]. One of the most widely used strategies for micropropagation is axillary bud proliferation, in which the nodal segments harboring axillary buds are cultured to regenerate the shoots [20,21]. This method is considered the easiest to apply and the most reliable way to produce clonal plants, which are genetically identical to the starting material. Organized meristems such as shoot tips and axillary buds are less prone to spontaneous genetic changes because meristems are more resistant to genetic changes than disorganized tissues [8,21,22,23,24]. An occasional somaclonal variation may occur in the plants regenerated from axillary buds at both the genetic and epigenetic levels. The underlying epigenetic mutations are often temporary in character, rarely maintain across generations, and the plants may revert to the typical phenotype relatively easily [8]. In contrast, genetic mutations are essentially irreversible and are likely to persist in the progeny of regenerated plants [25]. In this concern, the assessment of genetic uniformity of micropropagated plants can be performed using several DNA-based molecular markers [8,26]. Among various molecular markers available, simple sequence repeats (SSRs) markers have been widely and extensively used for the assessment of genetic homogeneity of micropropagated plants [26,27,28,29,30]. SSRs have advantages of high genomic abundance throughout the genome, codominant inheritance mode, high level of polymorphism, informativeness, high reproducibility, and robustness [27].

A variety of techniques for regeneration by organogenesis or somatic embryogenesis have been described in *H. annuus* and a few of its wild relatives [31,32,33,34,35,36,37,38,39,40,41,42,43]. The regeneration capacity by organogenesis is highly variable and depends upon many factors: genotype, specific media components, and the nature of explants, among others [32,33,34,38,39,40,41,42,43]. The application of micropropagation techniques is limited mainly by the difficulty of regenerating plants in a reproducible and efficient way [37,38,39,40,41,43]. Thus far, no complete studies have reported a protocol for an in vitro regeneration of *H. verticillatus*. To fill this gap, the goal of this project was to develop a rapid and efficient plant regeneration protocol for the multiplication of *H. verticillatus* genotypes by axillary bud proliferation, using nodal segments derived from greenhouse-grown plants as explants. This is especially important for this self-incompatible species because the plants derived from cross-pollination are characterized by a mixed genetic background. To achieve our goal, we investigated the in vitro response and regeneration capacity according to the genotype–culture condition interaction. We expected that both genotype and specific media components (concentration of cytokinin) would be the critical factors for the morphogenetic response. Developing the protocol included the following steps: axillary bud induction, shoot elongation, rooting, and acclimatization. Additionally, to assess the genetic homogeneity of the regenerated plants of *H. verticillatus*, highly polymorphic SSRs were used. To our knowledge, this is the first such report for this endangered species.

## 2. Results and Discussion

### 2.1. Induction of Axillary Bud Development

**Figure 1:**
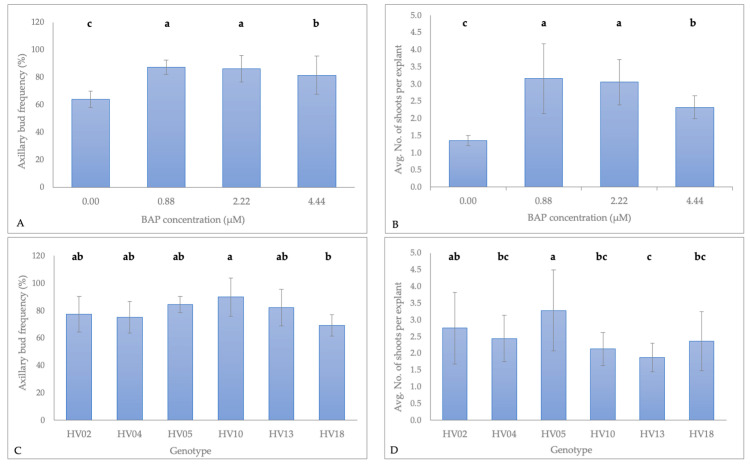
Effect of the 6-benzylaminopurine (BAP) concentration and the genotype on the axillary bud induction frequency (**A**,**C**) and the number of induced shoots per explant (**B**,**D**) in *H. verticillatus*. Each treatment (BAP concentration) and each genotype was represented by 96 and 64 nodal explants (each with 2 or 3 axillary buds), respectively. Error bars represent standard deviations (SD). Due to the lack of significance for the interaction BAP concentration–genotype on both the axillary bud induction frequency (*P* = 0.08) and the number of induced shoots per explant (*P* = 0.26), the main effects of both factors were investigated separately with one-way-ANOVAs and post-hoc Tukey honestly significant differences (HSD) tests (α = 0.05) (Table S1). The BAP concentration showed significant effects on both the axillary bud induction frequency (*p* < 0.01) and the number of shoots regenerated per explant (*p* < 0.05). The genotype showed a significant effect on both the axillary bud induction frequency (*p* < 0.01) and the number of shoots regenerated per nodal explant (*p* < 0.001). Lowercase letters above the bars represent grouping according to the Tukey tests post-one-way-ANOVAs at α = 0.05. HSD for the axillary bud induction frequency: 11.63% (BAP concentration) and 16.19% (genotype). HSD for the number of induced shoots per explant: 0.53 (BAP concentration) and 0.77 (genotype).

For a rapid in vitro clonal propagation of plants, normally dormant axillary buds are induced to elongate by cytokinins [22,44,45]. Among the various cytokinins tested in other studies, 6-benzylaminopurine (BAP) was the most widely used for the initiation and subsequent proliferation of shoots in a broad range of species [19,32,45,46,47,48]. Thus, the use of a ½-strength salts Murashige and Skoog medium (½ MS) and enhancing it with various concentrations of BAP (0.88 to 4.44 µM) were examined to assess the morphogenetic potential in the regeneration of six genotypes of *H. verticillatus* from nodal explants. After three–seven days of incubation on the induction media, swelling of the axillary buds was observed followed by bud break. From the responding buds, axillary shoots elongated within two weeks after the initial swelling. The axillary bud induction frequency (%) and number of shoots regenerated per nodal explant were significantly affected by the BAP concentration (Figure 1A,B) and genotype (Figure 1C,D) (Table S1).

**Figure 2:**
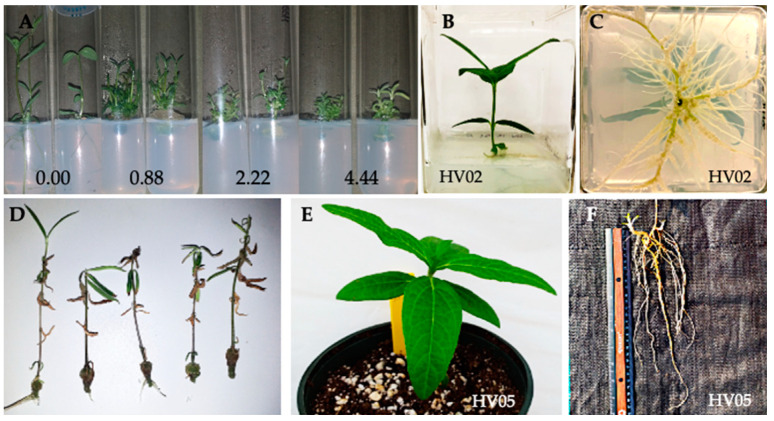
Micropropagation of *H. verticillatus*: shoot regeneration from nodal explants after 3 weeks of culturing on a ½ MS (induction medium) containing various concentration of BAP (**A**); regenerated shoots (HV02, HV05, HV10, HV13) showed healthy growth (**B**) and successful rooting (**C**) after 4 weeks culturing on an auxin-free ½ MS; regenerated shoots with physiological anomalies (HV04, HV18) and callus formed profusely at the cut end, the leaves turned brown, and occasionally the shoot apical meristem suspended growth and necrotized (**D**); plantlet acclimatization in Promix BX Mycorrhizae under greenhouse conditions: survived plantlets produced healthy new growth (**E**) with well-developed roots and fresh aboveground vegetative branches (**F**).

The absence of significance in the interaction between those two variables indicated that all induction media were able to induce both bud break and axillary shoot proliferation in nodal explants regardless of the genotypes tested. BAP in the medium significantly enhanced the axillary bud induction frequency as well as number of shoots when compared with the ½ MS medium without cytokinin. Higher bud induction frequency and number of shoots obtained from nodal segments exposed to BAP were a result of the higher number of induced shoot-buds and induction of shoots from axillary buds on the newly formed shoots (Figure 2A). Consistent with our findings, the positive impact of BAP on shoot induction from lateral buds has been reported in several plants [11,12,17,32,49,50].

The determination of the optimum level of cytokinins in the induction medium is considered critical for satisfactory shoot bud initiation and their proliferation because it depends on the species and cultivar, or the genotype cultured. In our study, of the three tested concentrations of BAP, the optimum concentration for bud break and shoot multiplication was either 0.88 or 2.22 µM. These concentrations of BAP stimulated an average of 3.2 ± 1.0 shoot-buds per nodal segment in 87.2 ± 5% of the axillary buds that responded to the induction of all six genotypes tested, and 3.1 ± 0.7 shoot-buds per nodal segment in 86.1 ± 9.5% of axillary buds, respectively. Increasing the BAP concentration to 4.44 µM did not improve the shoot response (81.4 ± 13.9%), and significantly reduced the number of induced shoots (2.3 ± 0.3) (Figure 1A,B). Increasing the concentration of BAP in the induction medium decreased the length of shoots (Figure 2A, and data not shown). A similar inhibitory effect of BAP beyond its optimum level on the axillary bud induction frequency, number of shoots per explants, and average shoot length has been well-documented in other plants [11,20,46,47,49,51,52,53,54,55,56,57]. A possible reason for the reduction in the regeneration potential was a detrimental effect of BAP at higher concentrations [58,59,60] or a formation of an excessive basal callus at the expense of shoot proliferation [12,56]. In our study, all explants incubated on media containing BAP produced a callus at the basal end of the nodal cuttings, regardless of the genotype (data not shown). Nevertheless, there was no clear relationship between the concentration of BAP and the callus intensity. This could indicate that this response might be influenced by other factors (e.g., physiological state or nodal stem localization) related to the endogenous level of the plant growth regulators (PGRs) in the plant tissue [61,62].

Although all media containing BAP were significantly more effective in inducing shoot development from the nodal explants than the medium devoid of cytokinin (Figure 1A,B), the lack of apical meristems in the nodal explants (simple sectioning and removal of the apical meristem) allowed the spontaneous production of axillary shoots. The BAP-free medium resulted in an overall relatively high shoot induction in 63.9 ± 5.9% of axillary buds with an average number of shoots at 1.4 ± 0.2 per explant. Each nodal stem segment formed two (occasionally three) buds, but in the majority of cultures, usually only one was able to grow, whereas the other(s) remained dormant. Such dominance of one bud over the others has also been observed in the micropropagated plants of a related species, *H. tuberosus* [63], and other species including *Dianthus* spp. [63], *Theobroma cacao* [64], and *Vitis vinifera* [65]. Furthermore, the lack of BAP in the induction medium promoted the growth of shoots displaying strong apical dominance that were more vigorous and grew faster than the ones induced by BAP (Figure 2A). Possible explanations of this include the absence of competition among the shoots and/or lack of exogenous cytokinin in the medium that maintained the original endogenous PGRs balance (the ratio of auxin to cytokinin).

The spontaneous induction of axillary shoots on the BAP-free medium observed in our study supports previous reports that apical stem removal halts the related dominance followed by changes in the endogenous PGRs in the nodal segments, and axillary buds start developing [66,67,68]. Similarly, the endogenous levels of PGRs were also sufficient to promote shoot growth from axillary buds in the nodal explants of other *Helianthus* species including *H. smithii* [33] and the hybrid of *H. eggertii* × *H. annuus* [39]. The PGR-free MS medium was also enough for the spontaneous production of axillary shoots from nodal explants of other species including *Mammillaria mathildae* [10], *Pinus* ssp. [69], *Sophora tonkinensis* [70], and *Vitis vinifera* [65]. The benefits of using BAP-free ½ MS for micropropagation of shoots from axillary buds include the limitation of somaclonal variations in the micropropagated plants [71]. To verify the regeneration efficiency from axillary buds on BAP-free ½ MS, 144 nodal explants (24 nodes per each genotype) derived from the in vitro regenerated shoots of six *H. verticillatus* plants were used for the subsequent propagation experiment on the same medium (data not shown). The results were congruent with our other findings and showed similar shoot bud initiation with 1.2 ± 0.1 of shoots per nodal segment in 58.2 ± 5.4% of axillary buds.

Genotype, among other factors, influences the in vitro organogenesis of various plant species, including *Helianthus* spp. [39,42]. Our micropropagation protocol enabled the regeneration of all tested genotypes from the nodal explants, however, significant differences were observed in the shoot formation frequency (*p* < 0.01) and the average number of shoots per explant (*p* < 0.001) among the tested genotypes (Figure 1C,D). The maximum response in the number of induced buds was recorded for HV10 (89.8 ± 14.0%). The majority of the remaining tested genotypes did not differ significantly in this regard except for HV18 (69.0 ± 7.8%), that reached the minimum response value of this parameter. The highest average number of shoots per explant was obtained for HV05 (3.3 ± 1.2), whereas the lowest was HV13 (1.9 ± 0.4). The differential morphogenetic response of the tested genotypes was likely due to the differences in the endogenous PGR levels among the individual *H. verticillatus* plants used in this study. Weber et al. [39] used apical meristems explants of the interspecific hybrids originating from crosses between *H. annuus* and nine others species of *Helianthus*, and concluded that a species regeneration capacity was significantly determined by the individual genotype. Variability in the organogenic responses from the axillary buds among genotypes was observed in many other species including *Rosa rugosa* [72], *Vigna unguiculata* [73], and *Lathyrus sativus* [74]. The observed variation in the morphogenetic response among the six tested plants of *H. verticillatus* warrants future studies for the levels of endogenous PGRs in the context of genotype and stem localization.

### 2.2. Rooting of Shoots Induced from the Axillary Buds

Successful rooting of the induced shoots is an essential requirement to facilitate their establishment in soil. The composition of the MS medium appears to be an important factor in influencing the rooting efficiency, and ½ MS was superior to the full-strength salts MS medium in this regard [25,51]. Many studies reported that the endogenous levels of auxins present in the tissue were sufficient to induce rooting in an auxin-free MS medium [10,11,50,75,76,77,78]. We used auxin-free ½ MS for the rooting of the induced shoots. Our aim was also to assess to what extent the addition of BAP in the induction medium impacted the shoot elongation inhibition and rooting efficiency in the subsequent rounds of subculture. Therefore, shoots regenerated from the nodal segments on all induction media used in this study were transferred directly to auxin-free ½ MS.

The factorial ANOVA showed a significant effect of the genotype and BAP concentration in the induction medium used for the initiation of axillary shoots from nodal stem segments, on the following parameters examined: stem length, root length, number of roots, and number of leaves. Moreover, all those parameters, except number of leaves, were significantly affected by the interaction between the genotype and BAP concentration (Table 1; Table S2).

The differences in growth and rooting efficiency were dependent on the composition of the induction medium and the genotype of the regenerated shoots. In general, the regenerated shoots had good growth regardless of the induction medium and the genotype (Figure 2B), except for two genotypes: HV04 and HV18.

Shoots derived from the axillary buds on the BAP-free ½ MS elongated rapidly, whereas those induced with any of the BAP treatments were less vigorous with shorter internodes and with significantly lower numbers of leaves (Table 1). Although shoots induced on the BAP-free ½ MS appeared more advanced in growth than those induced by BAP treatment, there were no significant differences in shoot and root lengths, except for the genotypes HV02 and HV05 (Table 1).

**Figure 3:**
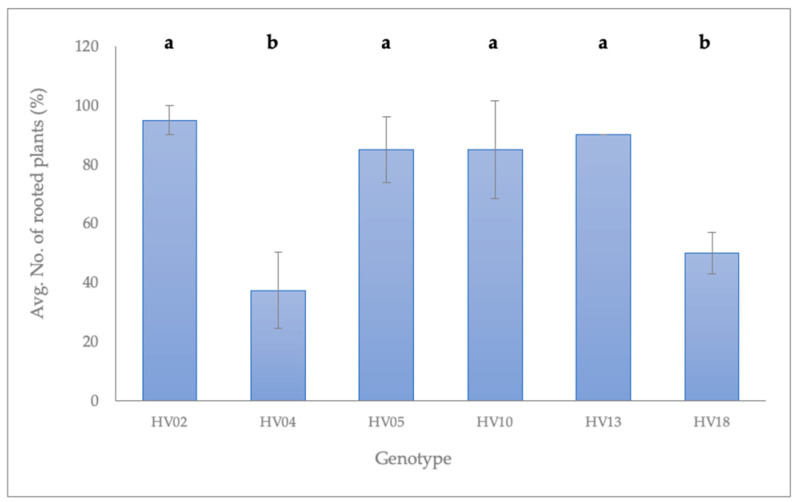
Frequency of rooting of *H*. *verticillatus* shoots recovered from the induced buds, scored after 4 weeks in culture on an auxin-free ½ MS (regeneration medium). Data are the average of raw counts obtained for shoots induced from the nodal segments on all induction media (*n* = 40). Error bars represent standard deviations (SD). Lowercase letters represent grouping according to the Tukey tests (HSD = 0.25) post-one-way-ANOVAs at α = 0.05.

Irrespective of the shoot origin (induction medium) or the genotype (other than HV04 and HV18), the vast majority of shoots were successfully rooted within four weeks after placement on auxin-free ½ MS (Figure 3).

The best rooting was achieved for HV02 (95 ± 5.0%), followed by HV13 (90 ± 0.0%), HV05 (87.5 ± 10.9%), and HV10 (85 ± 16.6%). Roots were well-developed with abundant secondary branching (Figure 2C). Rooting in the absence of exogenous auxins can be explained by the availability of endogenous auxins in the in vitro raised shoots. Similar observations have been reported for *N. rtanjensis* [11], *M. mathildae* [10], *Lavandula viridis* [76], *Dendrobium draconis* [78], *Phyllanthus stipulates* [75], and *V. vinifera* [77]. In all those species, shoots rooted spontaneously in an auxin-free MS medium. Shoots of the meristematic origin of the hybrid progenies involving four wild *Helianthus* species (*H. decapetalus*, *H. giganteus*, *H. mollis*, *H. strumosus*) rooted at an average frequency of 46–65%, when subcultured on an auxin-free regeneration medium [39].

The majority of shoots derived from two plants, HV04 and HV18, were characterized by poor growth as demonstrated by the genotype-dependent reaction in *H. verticillatus*. Leaves of these plantlets turned brown, and occasionally the apical meristem ceased growth and died (Figure 2D). A callus formed profusely at the cut end of those shoots regardless of the BAP concentration in the induction medium, although the callus production was more pronounced on induction media containing BAP. The number of plants with a callus formation at the stem base depended on the genotype and varied: 50.0 ± 7.1% (HV18) and 95 ± 8.7% (HV04). In contrast to the other *H. verticillatus* plants mentioned above, the rooting ability of the shoots regenerated from HV04 and HV18 was reduced. The average number of rooted shoots after four weeks of incubation on auxin-free ½ MS varied from 37.5 ± 13% on HV04 to 47.5 ± 13% for HV 18 (Figure 3). Importantly, shoots that exhibited any symptoms of the physiological anomalies recovered and grew normally after the apical tip excision and transfer to a fresh medium of the same composition. Rooting was promoted after one or several subcultures (Figure S1).

Our results indicated that the predisposition towards physiological anomalies was mainly genotype-dependent. One of the possible explanations for the reduced growth and rooting observed for the shoots derived from HV04 and HV18, may be the presence of abundant callus acting as a mechanical barrier to nutrient and water uptake [79]. Higher intensity of callus formation observed on these plants may be attributable to a disturbed balance of endogenous auxins and cytokinins, which consequently might affect the explant response to in vitro culturing conditions. Morphological anomalies and delay of rooting can be also triggered by a stress reaction during the adaptation of shoots to in vitro culturing conditions, which in turn might impose changes in their hormonal profiles.

### 2.3. Acclimatization

Rooted plantlets were transferred to a soilless mixture and successfully acclimatized to greenhouse environmental conditions. *Helianthus verticillatus* plants showed no special requirements for acclimatization or transplanting. Of the 120 plantlets transferred to the greenhouse (20 plantlets per genotype), 97% survived and produced healthy new growth (Figure 2E). The survival rate ranged from 95% to 100% for the genotypes evaluated. After eight weeks of acclimatization, roots were thick, long, and had secondary branching (Figure 2F). High survival rates have been also reported during the hardening and acclimatization stages of plants raised from the shoot tips in *H. annuus* [32] and its wild relatives [41]. There was no detectable variation either among the regenerated plants or between regenerants and their respective donor plants, with respect to morphological and growth characteristics (Figure S2).

### 2.4. Assessment of Genetic Fidelity of Micropropagated Plants

True-to-type clonal fidelity is one of the most important requirements in the micropropagation of any plant species. Plants regenerated from axillary buds show the lowest tendency for genetic variation [8,21]. However, the possibility of genetic variations arising during an in vitro process cannot be ruled out because tissue culture techniques are known to induce somaclonal variations in micropropagated plants [80]. Therefore, it is necessary to assess the genetic uniformity of the regenerated plants before incorporating a micropropagation protocol [15]. Morphological and physiological parameters have been used to ascertain the genetic fidelity of micropropagated plants in many species [81]; however, molecular markers are the most desirable tool for establishing the genetic uniformity and true-to-type nature of micropropagated plants [26,27,28,29,30].

**Figure 4:**
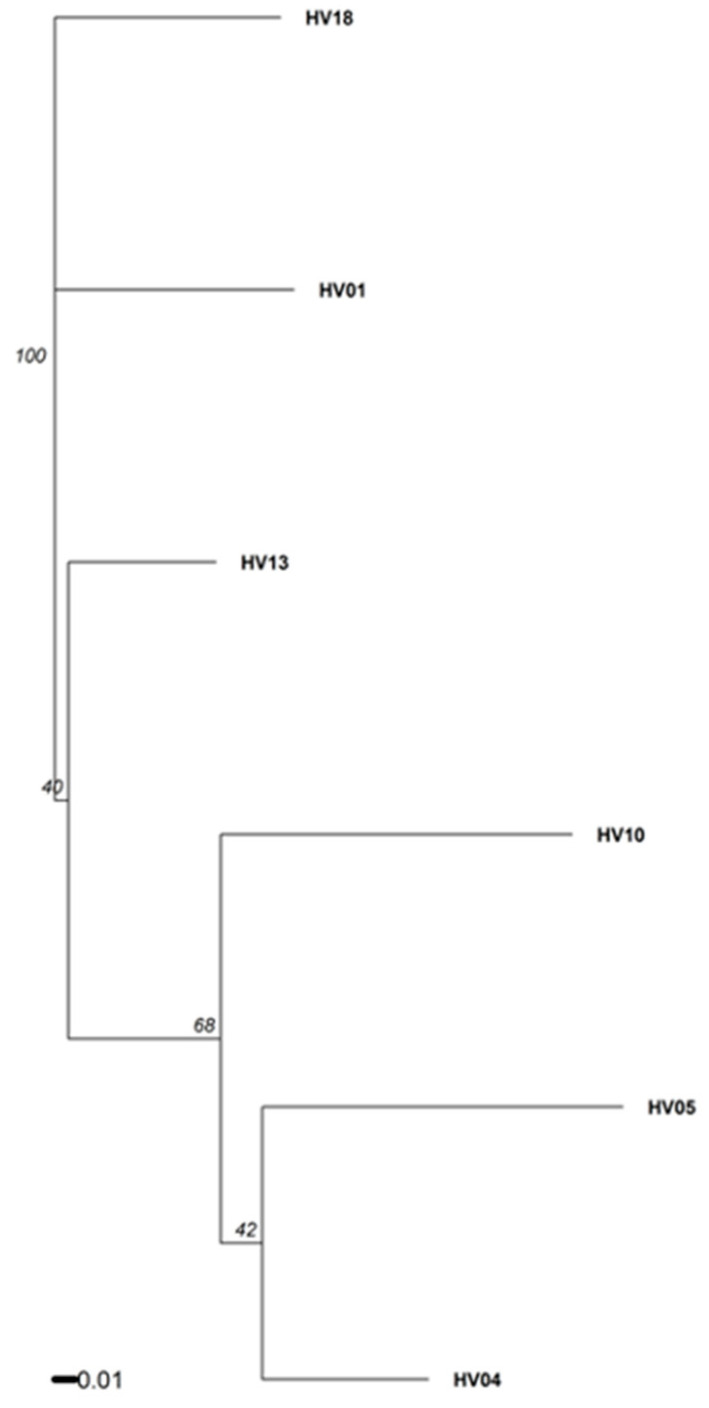
A dendrogram generated for data from seven polymorphic microsatellite markers (SSRs), illustrating the genetic distance among the donor plants. Plantlets propagated through axillary bud proliferation showed 100% similarity to their respective donor plants. The matrix of genetic distances was calculated in R version 3.6.1 using RStudio version 1.2.5019, and the package *poppr* version 2.8.3 [87] with Bruvo’s genetic distance [88] that regards the repeated motif lengths. Bootstrap support values for each split were calculated over 10,000 permutations of the dataset.

Out of the 14 SSR markers that were tested in this study, 7 were highly polymorphic among the donor plants, indicative of their genetic variation (Figure 4, Tables S3 and S4). Polymorphic SSRs produced 18 reproducible and clear bands ranging from 105 to 374 bp in size (Table S3). The number of alleles for each primer varied from two to four, with an average of 2.6 bands per SSR primer. Extraneous alleles were not detected between donor plants and their respective regenerants using all 14 SSRs (Table S4). A similarity matrix revealed that the pair-wise value between the donor plants and their regenerants was 1, indicating 100% identity, which confirmed the genetic uniformity and stability of regenerated plants of *H. verticillatus*.

Consistent with previous reports, our results demonstrate that direct plant regeneration through axillary shoot proliferation minimizes the chance of genetic instability and can be used as one of the safest techniques for the production of true-to-type plants [15,21,82,83,84,85,86].

## 3. Materials and Methods

### 3.1. Plant Materials and Explant Preparation

Six *H. verticillatus* plants exhibiting phenotypic variation in the morphology of leaves and stems were selected from the collection at the University of Tennessee, Knoxville, TN, USA (UT) as the source of explants for the tissue culture experiments. Based on genotyping using 7 polymorphic SSR markers, they were considered as six distinct genotypes (Figure 4). This collection was originally obtained from Madison Co. (TN) and Floyd Co. (GA) before *H. verticillatus* was classified as an endangered species [4].

Young 30 to 50 cm vegetative stems with several lateral buds were harvested from approximately 2-month-old or younger, freshly grown stalks grown in the greenhouse. After removing the leaves, stem segments and accompanying lateral buds were surface-sterilized with 70% ethanol for 2 min, followed with 40% (*v*/*v*) bleach solution (sodium hypochlorite 6%; Clorox, Oakland, CA, USA) containing two drops of Triton X-100 for 15 min, and finally washed five times with sterile distilled H_2_O. After removing the damaged edges, the stems were cut into 20 to 30 mm long segments, each containing two (rarely three) axillary buds. The experiment was carried-out in three independent series, each using two plants as the source of the nodal explants.

### 3.2. Media and Culture Conditions

Based on the protocols available for micropropagation of other species of *Helianthus* [33,41], ½-strength salts MS (PhytoTechnology Laboratories, Shawnee Mission, KS, USA) medium [89] was used as the basal medium. All media were supplemented with 1% (*w*/*v*) sucrose (Thermo Fisher Scientific, Pittsburgh, PA, USA) and 0.7% (*w*/*v*) phytoagar (PhytoTechnology Laboratories, Shawnee Mission, KS, USA). BAP was added to the medium in various concentrations and the pH was adjusted to 5.8 using 1 M NaOH, before autoclaving at 121 °C for 20 min. All cultures were incubated at 22 °C with a 12 h photoperiod provided by cool white fluorescent lamps.

### 3.3. Induction of Axillary Shoots from the Nodal Stem Segments

Nodal stem segments were cultured vertically in the 20 × 160 mm glass test tubes containing 20 mL of ½ MS or additionally supplemented with either 0.88, 2.22, or 4.44 µM BAP for axillary bud activation. Each treatment consisted of 16 culture tubes per genotype and each tube contained one nodal explant (total of 96 explants per treatment). After 3 weeks of incubation on the induction media, axillary bud induction frequency (percentage of induced axillary buds) and the number of shoots at least 5 mm in length per each responding explant were recorded.

### 3.4. Axillary Shoots Elongation and Rooting

Excised axillary shoots (20 to 25 mm long) derived from all induction media were transferred to Magenta GA7™ vessels (Magenta Corporation, Chicago, IL, USA) containing 50 mL of ½ MS without PGRs for elongation and spontaneous rooting. Each treatment (induction medium) was represented by 10 vessels per genotype and each contained one shoot (total of 60 shoots per treatment). After 4 weeks of culture, shoot and root length, number of roots produced per shoot, and number of leaves produced per shoot were evaluated. Shoots that did not form roots and/or showed poor growth were transferred repetitively to fresh ½ MS media vessels at 4-week intervals until roots emerged.

### 3.5. Acclimatization

For the acclimatization stage, in vitro rooted plantlets were washed with distilled H_2_O to remove adhering agar; then transplanted into plastic pots filled with Promix BX Mycorrhizae (Premier Horticulture Incorporation, Quakertown, PA, USA), under greenhouse conditions. The tops of the pots were covered with transparent plastic for one week to maintain high humidity. Each genotype was represented by 20 plantlets. The survival rate of the plantlets was evaluated after 8 weeks.

### 3.6. Assessment of Genetic Fidelity of Micropropagated Plants

For the genetic fidelity studies, total genomic DNA was extracted from fresh leaves of 42 randomly selected acclimatized plants and their donor plants (6 donor plants, 7 regenerated plants per each donor plant).

Samples were homogenized in micro-centrifuge tubes using zirconia beads/silica beads (BioSpec Products, Bartlesville, OK, USA) using a Bead Mill 24 (Fisher Scientific, Walther, MA, USA). DNA extraction was performed using a protocol of the E.Z.N.A. Plant DNA kit (Omega Bio-tek, Norcross, GA, USA) according to the manufacturer’s instructions. The concentration of DNA was measured using a NanoDrop ND-1000 spectrophotometer (NanoDrop Technologies, Wilmington, DE, USA) and stored at −20 °C. Fourteen tri- and tetra-repeat microsatellites, which had been selected for the genetic analysis of *H. verticillatus* in a previous report [90,91,92], were used for this study. DNA amplification was performed in 10 μL reactions with 4 ng genomic DNA and 0.25 μM of each primer, following the recommended protocol for AccuStart II PCR SuperMix (Quantabio, Beverly, MA, USA). Reactions were performed using the following touchdown PCR conditions: 95 °C for 3 min, followed by 10 cycles of 94 °C for 30 s, 65 °C lowering 1 °C per cycle to a final 55 °C for 30 s, then 72 °C for 45 s, another 30 cycles of 94 °C for 30 s, 55 °C for 30 s, 72 °C for 45 s, and a final elongation step at 72 °C for 20 min [91,92]. PCR products were separated using the QIAxcel Capillary Electrophoresis System (QIAGEN, Valencia, CA, USA) and sized with 25 to 500 base pair (bp) size markers and an internal 15/600 bp alignment marker [93]. Raw allele length data were then converted into discrete allelic classes using the program Flexibin [94]. The resulting data set was used for all further analyses. Out of the 14 tested markers [90,91,92], 7 allowed to distinguish the donor plants (Tables S3 and S4).

### 3.7. Statistical Analysis

Tissue culture data were analyzed using the analysis of variance (ANOVA) in the one- or two-way fashion, wherever applicable, using R version 3.6.1 in RStudio version 1.1.456, with the packages *car* version 3.0-5 and *agricolae* version 1.3-1 [95]. Subsequently, the honestly significant differences (HSD) at α = 0.05 were calculated using the same software. Standard errors and averages were calculated using MS Excel 2010 and R (ver. 3.6.1). The matrix of genetic distances was calculated in R version 3.6.1 using RStudio version 1.2.5019, and the package *poppr* version 2.8.3 [87]. Bruvo’s genetic distances that regard the repeated motif lengths were used for this analysis [88]. Bootstrap support values for each split in the dendrogram were calculated over 10,000 permutations of the dataset.

## 4. Conclusions

Our work showed that the nodal explants of *H. verticillatus* have a great morphogenetic potential for in vitro micropropagation through axillary bud proliferation. The protocol described in this study is an efficient, rapid, and simple technique for axillary bud initiation and shoot proliferation on a relatively simple nutrient medium without any PGRs. Notably, the addition of BAP to the induction medium enhanced the axillary bud induction frequency. As the requirement for exogenous PGRs depends directly on their endogenous levels in the plant tissue, which may vary with each individual plant genotype, organ, and the phase of growth, an adjustment of the concentration of cytokinins might be necessary for the less responsive genotypes.

Successful rooting of the induced shoots was achieved within four weeks after the transfer from the induction medium to fresh ½ MS, but the rooting efficiency was dependent on the plant’s genetic background. DNA analyses using highly polymorphic SSRs revealed 100% identity between the donor plants and their respective regenerants. This confirmed the genetic uniformity of the obtained plants. Hence, this technique can be applied for the generation of a large number of *H. verticillatus* plants well-suited for the conservation of germplasm or ecological and genetic studies.

## Figures and Tables

**Table 1:** Morphogenetic response of shoots of *H. verticillatus* regenerated and rooted on an auxin-free ½ MS.

The BAP Concentration (µM)	Plants	The Stem Length (cm) ^1^	The Root Length (cm) ^1^	The Number of Roots ^1^	The Number of Leaves ^2^
0.00	HV02	7.4 ± 1.6 a	9.1 ± 2.9 ab	4.6 ± 1.5 ab	6.5 ± 1.1
	HV04	4.9 ± 0.4 def	1.6 ± 0.4 e–j	1.8 ± 0.8 b–e	7.3 ± 1.5
	HV05	6.9 ± 1.4 b–f	9.6 ± 2.9 a–e	5.3 ± 1.3 abc	7.2 ± 1.0
	HV10	6.4 ± 1.4 b–f	8.4 ± 2.2 a–g	2.9 ± 0.9 b–f	7.8 ± 0.6
	HV13	4.0 ± 1.2 b–f	5.1 ± 1.8 hij	2.4 ± 0.9 def	8.9 ± 1.4
	HV18	6.7 ± 1.1 b–f	7.0 ± 2.0 j	3.8 ± 1.3 f	6.8 ± 1.0
	**Mean**	**6.1**	**6.8**	**3.5**	**7.4 A**
0.88	HV02	5.0 ± 2.2. c–f	6.0 ± 4.8 f–j	2.9 ± 0.8 def	8.9 ± 2.8
	HV04	4.6 ± 0.6 b–f	1.4 ± 0.8 hij	1.8 ± 0.8 def	8.4 ± 0.8
	HV05	3.2 ± 0.9 ab	2.3 ± 2.1 a	1.9 ± 0.9 a	9.2 ± 1.7
	HV10	5.9 ± 2.4 def	6.9 ± 2.4 d–j	2.0 ± 0.8 b–f	10.3 ± 1.3
	HV13	4.1 ± 1.3 ef	5.6 ± 1.5 f–j	2.1 ± 0.9 c–f	8.9 ± 1.0
	HV18	5.7 ± 1.2 f	3.1 ± 1.9 f–j	1.5 ± 1.0 c–f	8.7 ± 1.1
	**Mean**	**4.7**	**4.2**	**2.0**	**9.1 B**
2.22	HV02	4.6 ± 1.4 a–d	6.5 ± 2.0 d–j	3.3 ± 1.4 bcd	7.4 ± 1.4
	HV04	4.4 ± 0.5 a–f	6.2 ± 1.5 abc	2.3 ± 0.6 c–f	8.4 ± 0.7
	HV05	3.6 ± 0.5 a	3.1 ± 2.6 a–f	2.4 ± 1.3 b–e	9.4 ± 1.9
	HV10	7.4 ± 2.4 a–f	6.8 ± 3.4 a–d	3.4 ± 1.1 c–f	10.3 ± 1.8
	HV13	4.0 ± 0.5 d–f	5.0 ± 1.4 c–i	1.3 ± 0.7 c–f	8.0 ± 0.0
	HV18	5.8 ± 2.1 b–f	2.4 ± 2.3 c–j	2.0 ± 1.2 c–f	8.2 ± 1.5
	**Mean**	**5.0**	**5.0**	**2.5**	**8.6 B**
4.44	HV02	4.1 ± 1.0 def	2.6 ± 1.7 c–j	3.0 ± 1.4 c–f	8.5 ± 2.4
	HV04	4.7 ± 1.3 def	1.3 ± 1.1 b–h	2.0 ± 1.4 c–f	8.0 ± 0.7
	HV05	4.1 ± 1.1 abc	3.5 ± 3.1 c–j	3.1 ± 1.5 c–f	8.4 ± 0.7
	HV10	5.6 ± 1.4 a–e	3.4 ± 3.9 i–j	2.5 ± 0.8 e–f	9.0 ± 2.2
	HV13	4.7 ± 1.6 a–e	4.9 ± 1.6 hij	2.4 ± 1.1 def	9.6 ± 1.8
	HV18	5.9 ± 1.0 a–e	1.1 ± 0.4 g–j	4.3 ± 1.5 e–f	8.1 ± 0.3
	**Mean**	**4.8**	**2.8**	**2.9**	**8.6 B**

Data are the average (±SD) of raw counts obtained for shoots induced from the nodal segments on each induction medium (*n* = 10 per genotype and per BA concentration). ^1^ Lowercase letters represent grouping according to the Tukey tests post-two-way-ANOVAs (BAP concentration × genotype) at α = 0.05 (Table S2). HSD for the stem length: 2.41 cm. HSD for the root length: 4.31 cm. HSD for the number of roots: 2.27. ^2^ Due to the lack of significance for the interaction BAP concentration–genotype on the number of leaves (*P* = 0.05), the main effects of both factors were investigated separately with one-way-ANOVAs and post-hoc Tukey HSD tests (α = 0.05) (Table S2). Capital letters represent grouping according to the Tukey tests post-one-way-ANOVAs at α = 0.05. HSD for the BAP concentration: 1.06. HSD for the genotype: 0.76.

## Supplementary Materials

Supplementary Materials relating to this article are available online at https://www.mdpi.com/2223-7747/9/6/712/s1, Figure S1: Efficiency of rooting of shoots from six plants of *Helianthus verticillatus* after subsequent cycles of 4-week long subcultures on an auxin-free ½ MS (regeneration medium), Figure S2: Morphological comparison of donor plants (D) of *Helianthus verticillatus* and their respective regenerants (R1 to R3) grown in greenhouse, Table S1: Analysis of Variance (ANOVA) for the parameters examined for the induction of axillary shoots from the nodal stem segments of *Helianthus verticillatus* on a ½ MS containing various concentration of BAP (induction medium), Table S2: Analysis of Variance (ANOVA) for the parameters examined for the axillary shoots elongation and rooting of *Helianthus verticillatus* on an auxin-free ½ MS (regeneration medium), Table S3: Microsatellite loci used to examine the genetic uniformity of regenerants and their respective donor plants of *Helianthus verticillatus*, Table S4: Data obtained for genotyping of six donor plants of *Helianthus verticillatus* and their respective regenerants using SSRs.
