# Supplementary material for: In Vitro Propagation of an Endangered Helianthus verticillatus by Axillary Bud Proliferation
Source: Plants (Basel). 2020 Jun 3;9(6):712. doi: 10.3390/plants9060712 (PMC7356533; doi:10.3390/plants9060712)
Supplement: Supplementary file 1 [file plants-09-00712-s001.zip › plants-775981-supplementary/Figure S1.pdf]

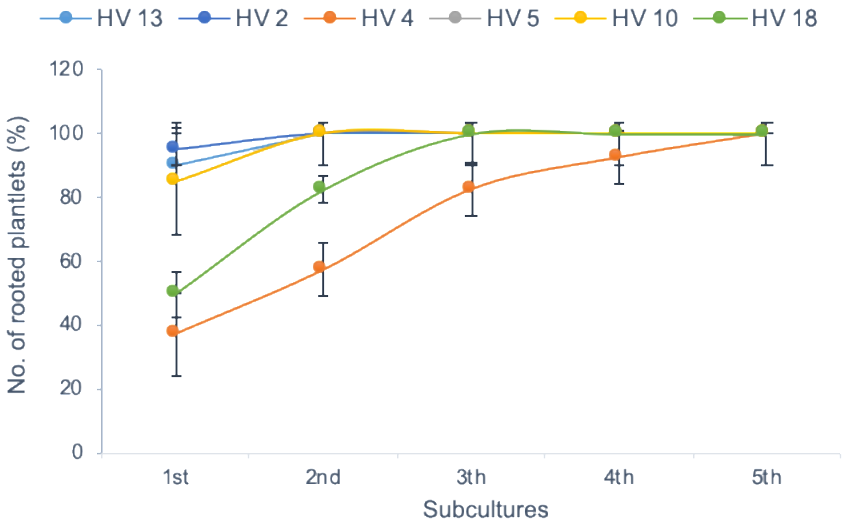

Figure S1. Efficiency of rooting of shoots from six plants of *Helianthus verticillatus* after subsequent cycles of 4-week long subcultures on an auxin-free ½ MS (regeneration medium). Data are the average of raw counts of rooted plantlets from the shoots induced from the nodal segments on all induction media (n = 40). Error bars represent standard deviation.
