# Supplementary material for: In Vitro Propagation of an Endangered Helianthus verticillatus by Axillary Bud Proliferation
Source: Plants (Basel). 2020 Jun 3;9(6):712. doi: 10.3390/plants9060712 (PMC7356533; doi:10.3390/plants9060712)
Supplement: Supplementary file 1 [file plants-09-00712-s001.zip › plants-775981-supplementary/Figure S2.pdf]

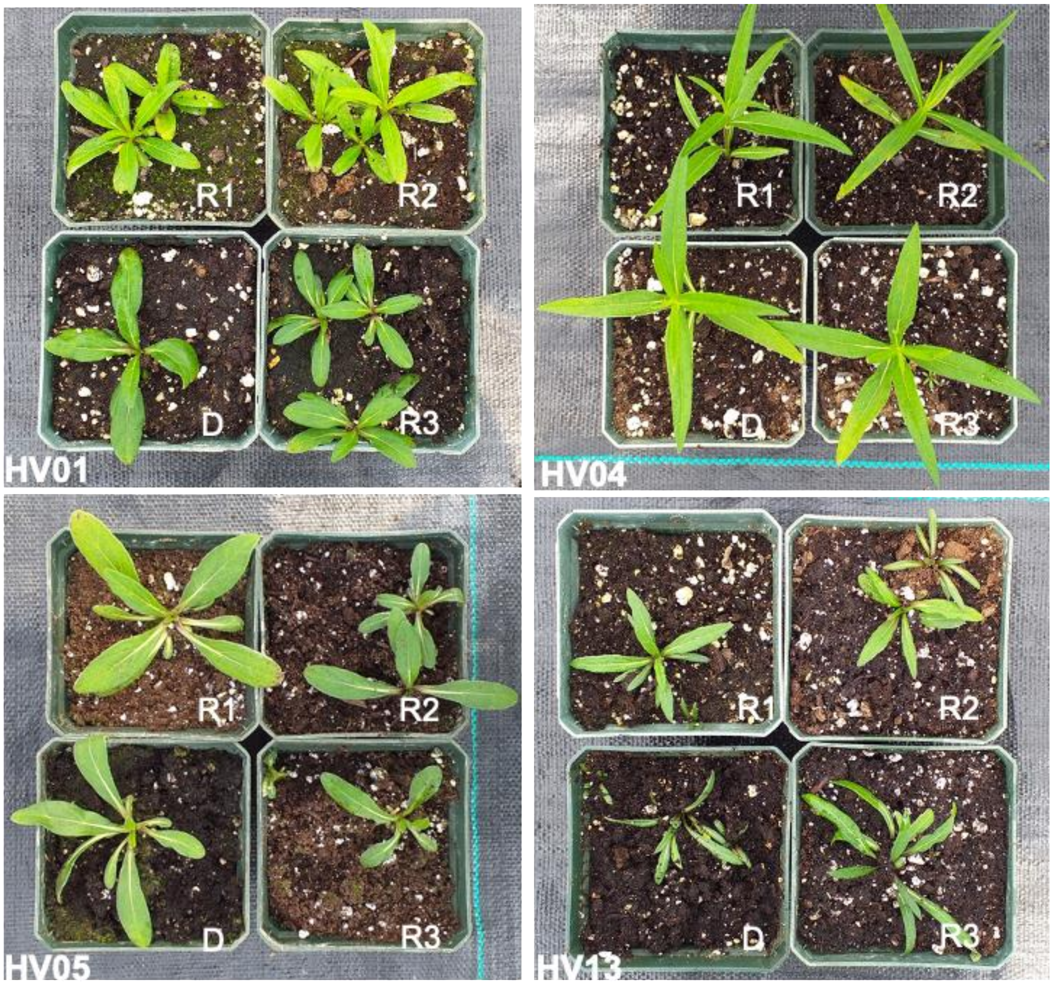

Figure S2. Morphological comparison of donor plants (D) of *Helianthus verticillatus* and their respective regenerants (R1 to R3) grown in greenhouse. There were no noticeable phenotypical differences among regenerated plants as well as between them and their respective donor plants. The propagated plants were characterized by the same leaves' morphology as donor plants.
