# Supplementary material for: In Vitro Propagation of an Endangered Helianthus verticillatus by Axillary Bud Proliferation
Source: Plants (Basel). 2020 Jun 3;9(6):712. doi: 10.3390/plants9060712 (PMC7356533; doi:10.3390/plants9060712)
Supplement: Supplementary file 1 [file plants-09-00712-s001.zip › plants-775981-supplementary/Table S1.pdf]

Table S1. Analysis of Variance (ANOVA) for the parameters examined for the induction of axillary shoots from the nodal stem segments of *Helianthus verticillatus* on a ½ MS containing various concentration of BAP (induction medium).

Tested Parameters	Variation Source	Sum of Squares	F Value	P [Pr(>F)]
Axillary bud induction frequency	Genotype	17492	3.5232	0.003997 **
	BAP concentration	7281	7.3323	0.007085 **
	BAP × genotype	9779	1.9696	0.082365 ^{ns}
Number of shoots per explants	Genotype	78.26	6.8421	4.015e-06 ***
	BAP concentration	13.50	5.9023	0.01559 *
	BAP × genotype	14.90	1.3022	0.26220 ^{ns}

*—significant at $p < 0.05$; **—significant at $p < 0.01$; ***—significant at $p < 0.001$; ^{ns}—not significant.
