# Supplementary material for: In Vitro Propagation of an Endangered Helianthus verticillatus by Axillary Bud Proliferation
Source: Plants (Basel). 2020 Jun 3;9(6):712. doi: 10.3390/plants9060712 (PMC7356533; doi:10.3390/plants9060712)
Supplement: Supplementary file 1 [file plants-09-00712-s001.zip › plants-775981-supplementary/Table S2.pdf]

Table S2. Analysis of Variance (ANOVA) for the parameters examined for the axillary shoots elongation and rooting of *Helianthus verticillatus* on an auxin-free ½ MS (regeneration medium).

Tested Parameters	Variation Source	Sum of Squares	F Value	P [Pr(>F)]
Stem length	Genotype	141.20	11.1862	1.179e-09 ***
	BAP concentration	37.86	14.9962	0.0001408 ***
	BAP × genotype	36.27	2.8736	0.0154555 *
Root length	Genotype	942.15	20.3398	<2.2e-16 ***
	BAP concentration	67.12	7.2447	0.007638 **
	BAP × genotype	165.73	3.5778	0.003907 **
Number of roots	Genotype	177.65	16.7613	4.265e-14 ***
	BAP concentration	45.20	21.3214	4.265e-14 ***
	BAP × genotype	37.25	3.5142	0.00443 **
Number of leaves	Genotype	71.90	6.0214	2.956e-05 ***
	BAP concentration	63.45	26.5688	5.509e-07 ***
	BAP × genotype	26.80	2.2446	0.05085 ^{ns}
No of rooted plantlets	Genotype	12.421	16.342	8.96e-14 ***
	BAP concentration	0.003	0.018	0.8946 ^{ns}
	BAP × genotype	0.313	0.412	0.8399 ^{ns}

*—significant at $p < 0.05$; **—significant at $p < 0.01$; ***—significant at $p < 0.001$; ^{ns}—not significant.
