# Supplementary material for: In Vitro Propagation of an Endangered Helianthus verticillatus by Axillary Bud Proliferation
Source: Plants (Basel). 2020 Jun 3;9(6):712. doi: 10.3390/plants9060712 (PMC7356533; doi:10.3390/plants9060712)
Supplement: Supplementary file 1 [file plants-09-00712-s001.zip › plants-775981-supplementary/Table S3.pdf]

Table S3. Microsatellite loci used to examine the genetic uniformity of regenerants and their respective donor plants of *Helianthus verticillatus*.

Core Loci [A]	Renamed Loci [B]	Forward and Reverse Primers 5′-3′	Repeat Motif	Number of Alleles	Allele Size Range (bp)
BL0006	HV006	F: CATGGGTGATCAATGGAGTG<br>R: CGGCACATAACAAGTGCTTC	$(gtga)_3$	2	230–265
BL0013	HV012	F: CGAGACGGTTAAGAGCTTGC<br>R: GGTGTACAACCAACTCACACC	$(gtta)_3$	4	337–349
BL0022	HV017	F: ACTTACCGTTGCATTTGGTG<br>R: GCTTATCCCTAGAACACGATTACAG	$(taa)_4$	2	105–108
BL0015	HV024	F: AATTGGAGCGGATGGTATTG<br>R: AATATCTCTTATTTCAATAGTCCAACG	$(atg)_4$	1	360
BL0019	HV026	F: GAGTCCTGGCCTGAACAGAG<br>R: CAAACTGCAATGTACCTTCTTGAC	$(gaaa)_3$	1	296
BL0024	HV028	F: CTCCCGCACTTCAAGCTAAC<br>R: CATACACCTTTGCGGTTTCC	$(gtaa)_3$	1	119
BL0031	HV031	F: CCGGAAGATAACGACGAGTG<br>R: TCCATCGCTTTCCCTAAATC	$(gac)_4$	1	424
BL0033	HV033	F: GGGAGTTACACGCCTCCAG<br>R: CACAACCATACGCCATCAAG	$(cac)_4$	2	265–268
BL0034	HV034	F: GGTCGTCTACTACGGCTTCG<br>R: TAACCGAACGACCATTCTTC	$(tgtt)_4$	3	154–162
BL0037	HV037	F: GGTTAGGGTGAGGGTGGTG<br>R: AAGCCATAGTAAGTTCCTCTTACAAAC	$(tgca)_3$	3	152–176
BL0041	HV041	F: ACATTTGGACGTTTGGAAGC<br>R: TCCATCGAGATGTTGACACG	$(ctt)_4$	1	190
BL0042	HV042	F: GGTTACAACGGTGGAAGTCG<br>R: TCCGGTTCACCAATTCATTC	$(ggc)_4$	2	368–374
BL0046	HV046	F: GAACCAACACAACCAAATCC<br>R: TGTCGCTTCAACGCATAAAC	$(aaca)_3$	1	317
BL0048	HV048	F: TTGTGGAGACGGTGAATGAG<br>R: TAACCGAACGACCATTCTTC	$(gaa)_4$	1	224

[A]—All core loci taken from Pashley et al. [91]; [B]—Loci renamed by Edwards et al. [5].
