# Supplementary material for: In Vitro Propagation of an Endangered Helianthus verticillatus by Axillary Bud Proliferation
Source: Plants (Basel). 2020 Jun 3;9(6):712. doi: 10.3390/plants9060712 (PMC7356533; doi:10.3390/plants9060712)
Supplement: Supplementary file 1 [file plants-09-00712-s001.zip › plants-775981-supplementary/Table S4.pdf]

**Table S4.** Data obtained for genotyping of six donor plants of *Helianthus verticillatus* and their respective regenerants using SSRs.

| Plant | Product Size (bp) | | | | | | | | | | | | | |
|---|---|---|---|---|---|---|---|---|---|---|---|---|---|---|
| | HV006 | | HV012 | | HV017 | | HV033 | | HV034 | | HV037 | | HV042 | |
| **HV01.D** | **265** | **265** | **337** | **337** | **108** | **108** | **265** | **265** | **154** | **154** | **160** | **176** | **368** | **368** |
| HV01.R1 | 265 | 265 | 337 | 337 | 108 | 108 | 265 | 265 | 154 | 154 | 160 | 176 | 368 | 368 |
| HV01.R2 | 265 | 265 | 337 | 337 | 108 | 108 | 265 | 265 | 154 | 154 | 160 | 176 | 368 | 368 |
| HV01.R3 | 265 | 265 | 337 | 337 | 108 | 108 | 265 | 265 | 154 | 154 | 160 | 176 | 368 | 368 |
| HV01.R4 | 265 | 265 | 337 | 337 | 108 | 108 | 265 | 265 | 154 | 154 | 160 | 176 | 368 | 368 |
| HV01.R5 | 265 | 265 | 337 | 337 | 108 | 108 | 265 | 265 | 154 | 154 | 160 | 176 | 368 | 368 |
| HV01.R6 | 265 | 265 | 337 | 337 | 108 | 108 | 265 | 265 | 154 | 154 | 160 | 176 | 368 | 368 |
| HV01.R7 | 265 | 265 | 337 | 337 | 108 | 108 | 265 | 265 | 154 | 154 | 160 | 176 | 368 | 368 |
| **HV04.D** | **265** | **265** | **341** | **349** | **105** | **105** | **265** | **265** | **154** | **154** | **152** | **160** | **368** | **374** |
| HV04.R1 | 265 | 265 | 341 | 349 | 105 | 105 | 265 | 265 | 154 | 154 | 152 | 160 | 368 | 374 |
| HV04.R2 | 265 | 265 | 341 | 349 | 105 | 105 | 265 | 265 | 154 | 154 | 152 | 160 | 368 | 374 |
| HV04.R3 | 265 | 265 | 341 | 349 | 105 | 105 | 265 | 265 | 154 | 154 | 152 | 160 | 368 | 374 |
| HV04.R4 | 265 | 265 | 341 | 349 | 105 | 105 | 265 | 265 | 154 | 154 | 152 | 160 | 368 | 374 |
| HV04.R5 | 265 | 265 | 341 | 349 | 105 | 105 | 265 | 265 | 154 | 154 | 152 | 160 | 368 | 374 |
| HV04.R6 | 265 | 265 | 341 | 349 | 105 | 105 | 265 | 265 | 154 | 154 | 152 | 160 | 368 | 374 |
| HV04.R7 | 265 | 265 | 341 | 349 | 105 | 105 | 265 | 265 | 154 | 154 | 152 | 160 | 368 | 374 |
| **HV05.D** | **265** | **265** | **341** | **341** | **108** | **108** | **265** | **265** | **158** | **158** | **160** | **176** | **368** | **374** |
| HV05.R1 | 265 | 265 | 341 | 341 | 108 | 108 | 265 | 265 | 158 | 158 | 160 | 176 | 368 | 374 |
| HV05.R2 | 265 | 265 | 341 | 341 | 108 | 108 | 265 | 265 | 158 | 158 | 160 | 176 | 368 | 374 |
| HV05.R3 | 265 | 265 | 341 | 341 | 108 | 108 | 265 | 265 | 158 | 158 | 160 | 176 | 368 | 374 |
| HV05.R4 | 265 | 265 | 341 | 341 | 108 | 108 | 265 | 265 | 158 | 158 | 160 | 176 | 368 | 374 |
| HV05.R5 | 265 | 265 | 341 | 341 | 108 | 108 | 265 | 265 | 158 | 158 | 160 | 176 | 368 | 374 |
| HV05.R6 | 265 | 265 | 341 | 341 | 108 | 108 | 265 | 265 | 158 | 158 | 160 | 176 | 368 | 374 |
| HV05.R7 | 265 | 265 | 341 | 341 | 108 | 108 | 265 | 265 | 158 | 158 | 160 | 176 | 368 | 374 |

D—donor plant; R1 to R7—regenerants obtained through axillary bud proliferation.

**Table S4.** Data obtained for genotyping of six donor plants of *Helianthus verticillatus* and their respective regenerants using SSRs (continue).

| **Plant** | **Product Size (bp)** | | | | | | | | | | | | | |
|---|---|---|---|---|---|---|---|---|---|---|---|---|---|---|
| | **HV006** | | **HV012** | | **HV017** | | **HV033** | | **HV034** | | **HV037** | | **HV042** | |
| **HV10.D** | **265** | **265** | **345** | **345** | **108** | **108** | **268** | **268** | **154** | **154** | **152** | **160** | **374** | **374** |
| HV10.R1 | 265 | 265 | 345 | 345 | 108 | 108 | 268 | 268 | 154 | 154 | 152 | 160 | 374 | 374 |
| HV10.R2 | 265 | 265 | 345 | 345 | 108 | 108 | 268 | 268 | 154 | 154 | 152 | 160 | 374 | 374 |
| HV10.R3 | 265 | 265 | 345 | 345 | 108 | 108 | 268 | 268 | 154 | 154 | 152 | 160 | 374 | 374 |
| HV10.R4 | 265 | 265 | 345 | 345 | 108 | 108 | 268 | 268 | 154 | 154 | 152 | 160 | 374 | 374 |
| HV10.R5 | 265 | 265 | 345 | 345 | 108 | 108 | 268 | 268 | 154 | 154 | 152 | 160 | 374 | 374 |
| HV10.R6 | 265 | 265 | 345 | 345 | 108 | 108 | 268 | 268 | 154 | 154 | 152 | 160 | 374 | 374 |
| HV10.R7 | 265 | 265 | 345 | 345 | 108 | 108 | 268 | 268 | 154 | 154 | 152 | 160 | 374 | 374 |
| **HV13.D** | **230** | **265** | **341** | **349** | **105** | **105** | **268** | **268** | **154** | **162** | **152** | **176** | **368** | **374** |
| HV13.R1 | 230 | 265 | 341 | 349 | 105 | 105 | 268 | 268 | 154 | 162 | 152 | 176 | 368 | 374 |
| HV13.R2 | 230 | 265 | 341 | 349 | 105 | 105 | 268 | 268 | 154 | 162 | 152 | 176 | 368 | 374 |
| HV13.R3 | 230 | 265 | 341 | 349 | 105 | 105 | 268 | 268 | 154 | 162 | 152 | 176 | 368 | 374 |
| HV13.R4 | 230 | 265 | 341 | 349 | 105 | 105 | 268 | 268 | 154 | 162 | 152 | 176 | 368 | 374 |
| HV13.R5 | 230 | 265 | 341 | 349 | 105 | 105 | 268 | 268 | 154 | 162 | 152 | 176 | 368 | 374 |
| HV13.R6 | 230 | 265 | 341 | 349 | 105 | 105 | 268 | 268 | 154 | 162 | 152 | 176 | 368 | 374 |
| HV13.R7 | 230 | 265 | 341 | 349 | 105 | 105 | 268 | 268 | 154 | 162 | 152 | 176 | 368 | 374 |
| **HV18.D** | **265** | **265** | **341** | **345** | **108** | **108** | **265** | **265** | **154** | **162** | **152** | **176** | **368** | **368** |
| HV18.R1 | 265 | 265 | 341 | 345 | 108 | 108 | 265 | 265 | 154 | 162 | 152 | 176 | 368 | 368 |
| HV18.R2 | 265 | 265 | 341 | 345 | 108 | 108 | 265 | 265 | 154 | 162 | 152 | 176 | 368 | 368 |
| HV18.R3 | 265 | 265 | 341 | 345 | 108 | 108 | 265 | 265 | 154 | 162 | 152 | 176 | 368 | 368 |
| HV18.R4 | 265 | 265 | 341 | 345 | 108 | 108 | 265 | 265 | 154 | 162 | 152 | 176 | 368 | 368 |
| HV18.R5 | 265 | 265 | 341 | 345 | 108 | 108 | 265 | 265 | 154 | 162 | 152 | 176 | 368 | 368 |
| HV18.R6 | 265 | 265 | 341 | 345 | 108 | 108 | 265 | 265 | 154 | 162 | 152 | 176 | 368 | 368 |
| HV18.R7 | 265 | 265 | 341 | 345 | 108 | 108 | 265 | 265 | 154 | 162 | 152 | 176 | 368 | 368 |

D—donor plant; R1 to R7—regenerants obtained through axillary bud proliferation.

**Table S4.** Data obtained for genotyping of six donor plants of *Helianthus verticillatus* and their respective regenerants using SSRs (continue).

| Plant | Product Size (bp) | | | | | | | | | | | | | |
|---|---|---|---|---|---|---|---|---|---|---|---|---|---|---|
| | HV024 | | HV026 | | HV028 | | HV031 | | HV041 | | HV046 | | HV048 | |
| **HV01.D** | **360** | **360** | **296** | **296** | **119** | **119** | **424** | **424** | **190** | **190** | **317** | **317** | **224** | **224** |
| HV01.R1 | 360 | 360 | 296 | 296 | 119 | 119 | 424 | 424 | 190 | 190 | 317 | 317 | 224 | 224 |
| HV01.R2 | 360 | 360 | 296 | 296 | 119 | 119 | 424 | 424 | 190 | 190 | 317 | 317 | 224 | 224 |
| HV01.R3 | 360 | 360 | 296 | 296 | 119 | 119 | 424 | 424 | 190 | 190 | 317 | 317 | 224 | 224 |
| HV01.R4 | 360 | 360 | 296 | 296 | 119 | 119 | 424 | 424 | 190 | 190 | 317 | 317 | 224 | 224 |
| HV01.R5 | 360 | 360 | 296 | 296 | 119 | 119 | 424 | 424 | 190 | 190 | 317 | 317 | 224 | 224 |
| HV01.R6 | 360 | 360 | 296 | 296 | 119 | 119 | 424 | 424 | 190 | 190 | 317 | 317 | 224 | 224 |
| HV01.R7 | 360 | 360 | 296 | 296 | 119 | 119 | 424 | 424 | 190 | 190 | 317 | 317 | 224 | 224 |
| **HV04.D** | **360** | **360** | **296** | **296** | **119** | **119** | **424** | **424** | **190** | **190** | **317** | **317** | **224** | **224** |
| HV04.R1 | 360 | 360 | 296 | 296 | 119 | 119 | 424 | 424 | 190 | 190 | 317 | 317 | 224 | 224 |
| HV04.R2 | 360 | 360 | 296 | 296 | 119 | 119 | 424 | 424 | 190 | 190 | 317 | 317 | 224 | 224 |
| HV04.R3 | 360 | 360 | 296 | 296 | 119 | 119 | 424 | 424 | 190 | 190 | 317 | 317 | 224 | 224 |
| HV04.R4 | 360 | 360 | 296 | 296 | 119 | 119 | 424 | 424 | 190 | 190 | 317 | 317 | 224 | 224 |
| HV04.R5 | 360 | 360 | 296 | 296 | 119 | 119 | 424 | 424 | 190 | 190 | 317 | 317 | 224 | 224 |
| HV04.R6 | 360 | 360 | 296 | 296 | 119 | 119 | 424 | 424 | 190 | 190 | 317 | 317 | 224 | 224 |
| HV04.R7 | 360 | 360 | 296 | 296 | 119 | 119 | 424 | 424 | 190 | 190 | 317 | 317 | 224 | 224 |
| **HV05.D** | **360** | **360** | **296** | **296** | **119** | **119** | **424** | **424** | **190** | **190** | **317** | **317** | **224** | **224** |
| HV05.R1 | 360 | 360 | 296 | 296 | 119 | 119 | 424 | 424 | 190 | 190 | 317 | 317 | 224 | 224 |
| HV05.R2 | 360 | 360 | 296 | 296 | 119 | 119 | 424 | 424 | 190 | 190 | 317 | 317 | 224 | 224 |
| HV05.R3 | 360 | 360 | 296 | 296 | 119 | 119 | 424 | 424 | 190 | 190 | 317 | 317 | 224 | 224 |
| HV05.R4 | 360 | 360 | 296 | 296 | 119 | 119 | 424 | 424 | 190 | 190 | 317 | 317 | 224 | 224 |
| HV05.R5 | 360 | 360 | 296 | 296 | 119 | 119 | 424 | 424 | 190 | 190 | 317 | 317 | 224 | 224 |
| HV05.R6 | 360 | 360 | 296 | 296 | 119 | 119 | 424 | 424 | 190 | 190 | 317 | 317 | 224 | 224 |
| HV05.R7 | 360 | 360 | 296 | 296 | 119 | 119 | 424 | 424 | 190 | 190 | 317 | 317 | 224 | 224 |

D—donor plant; R1 to R7—regenerants obtained through axillary bud proliferation.

**Table S4.** Data obtained for genotyping of six donor plants of *Helianthus verticillatus* and their respective regenerants using SSRs (continue).

| **Plant** | **Product Size (bp)** | | | | | | | | | | | | | |
|---|---|---|---|---|---|---|---|---|---|---|---|---|---|---|
| | **HV024** | | **HV026** | | **HV028** | | **HV031** | | **HV041** | | **HV046** | | **HV048** | |
| **HV10.D** | **360** | **360** | **296** | **296** | **119** | **119** | **424** | **424** | **190** | **190** | **317** | **317** | **224** | **224** |
| HV10.R1 | 360 | 360 | 296 | 296 | 119 | 119 | 424 | 424 | 190 | 190 | 317 | 317 | 224 | 224 |
| HV10.R2 | 360 | 360 | 296 | 296 | 119 | 119 | 424 | 424 | 190 | 190 | 317 | 317 | 224 | 224 |
| HV10.R3 | 360 | 360 | 296 | 296 | 119 | 119 | 424 | 424 | 190 | 190 | 317 | 317 | 224 | 224 |
| HV10.R4 | 360 | 360 | 296 | 296 | 119 | 119 | 424 | 424 | 190 | 190 | 317 | 317 | 224 | 224 |
| HV10.R5 | 360 | 360 | 296 | 296 | 119 | 119 | 424 | 424 | 190 | 190 | 317 | 317 | 224 | 224 |
| HV10.R6 | 360 | 360 | 296 | 296 | 119 | 119 | 424 | 424 | 190 | 190 | 317 | 317 | 224 | 224 |
| HV10.R7 | 360 | 360 | 296 | 296 | 119 | 119 | 424 | 424 | 190 | 190 | 317 | 317 | 224 | 224 |
| **HV13.D** | **360** | **360** | **296** | **296** | **119** | **119** | **424** | **424** | **190** | **190** | **317** | **317** | **224** | **224** |
| HV13.R1 | 360 | 360 | 296 | 296 | 119 | 119 | 424 | 424 | 190 | 190 | 317 | 317 | 224 | 224 |
| HV13.R2 | 360 | 360 | 296 | 296 | 119 | 119 | 424 | 424 | 190 | 190 | 317 | 317 | 224 | 224 |
| HV13.R3 | 360 | 360 | 296 | 296 | 119 | 119 | 424 | 424 | 190 | 190 | 317 | 317 | 224 | 224 |
| HV13.R4 | 360 | 360 | 296 | 296 | 119 | 119 | 424 | 424 | 190 | 190 | 317 | 317 | 224 | 224 |
| HV13.R5 | 360 | 360 | 296 | 296 | 119 | 119 | 424 | 424 | 190 | 190 | 317 | 317 | 224 | 224 |
| HV13.R6 | 360 | 360 | 296 | 296 | 119 | 119 | 424 | 424 | 190 | 190 | 317 | 317 | 224 | 224 |
| HV13.R7 | 360 | 360 | 296 | 296 | 119 | 119 | 424 | 424 | 190 | 190 | 317 | 317 | 224 | 224 |
| **HV18.D** | **360** | **360** | **296** | **296** | **119** | **119** | **424** | **424** | **190** | **190** | **317** | **317** | **224** | **224** |
| HV18.R1 | 360 | 360 | 296 | 296 | 119 | 119 | 424 | 424 | 190 | 190 | 317 | 317 | 224 | 224 |
| HV18.R2 | 360 | 360 | 296 | 296 | 119 | 119 | 424 | 424 | 190 | 190 | 317 | 317 | 224 | 224 |
| HV18.R3 | 360 | 360 | 296 | 296 | 119 | 119 | 424 | 424 | 190 | 190 | 317 | 317 | 224 | 224 |
| HV18.R4 | 360 | 360 | 296 | 296 | 119 | 119 | 424 | 424 | 190 | 190 | 317 | 317 | 224 | 224 |
| HV18.R5 | 360 | 360 | 296 | 296 | 119 | 119 | 424 | 424 | 190 | 190 | 317 | 317 | 224 | 224 |
| HV18.R6 | 360 | 360 | 296 | 296 | 119 | 119 | 424 | 424 | 190 | 190 | 317 | 317 | 224 | 224 |
| HV18.R7 | 360 | 360 | 296 | 296 | 119 | 119 | 424 | 424 | 190 | 190 | 317 | 317 | 224 | 224 |

D—donor plant; R1 to R7—regenerants obtained through axillary bud proliferation.
